# Nursing radiation-protection compliance and 30-day wound and pin-site adverse events after supracondylar humerus fracture fixation in children

**DOI:** 10.3389/fped.2026.1902962

**Published:** 2026-10-15

**Authors:** Yuechao Huang, Ziye Huang, Wenlu Jin

**Affiliations:** OuJiangkou Operating Room, The Second Affiliated Hospital of Wenzhou Medical University, Wenzhou, Zhejiang, China

**Keywords:** fluoroscopy, nursing compliance, pediatric orthopedics, pin-site complications, radiation protection, retrospective cohort study, supracondylar humerus fracture, surgical wound infection

## Abstract

**Background:**

Fluoroscopy is integral to operative fixation of supracondylar humerus fractures in children, but case-level nursing radiation-protection compliance (NRPC) has not been well characterised as a potential marker of broader perioperative process quality. We examined the association between NRPC and early wound- and pin-site–related outcomes.

**Methods:**

We conducted a single-centre retrospective cohort study of children aged <14 years who underwent fluoroscopy-assisted operative fixation for supracondylar humerus fractures at The Second Affiliated Hospital of Wenzhou Medical University, China, between 2018 and 2023. NRPC was assessed using three binary components reflecting recent formal radiation-protection training, absence of radiation-protection–related quality-control deductions, and documented implementation of radiation-protection measures during the index procedure. Components were summed to a 0–3 score and categorised as Low (0–1) or High (2–3) NRPC. The primary outcome was any wound- or pin-site–related adverse event within 30 days. The primary multivariable logistic regression model adjusted for calendar year, Gartland classification, operative duration, and emergency surgical status. Firth penalized regression, ordinal-score analyses, and separate nurse- and surgeon-clustered mixed-effects models were performed as sensitivity analyses.

**Results:**

Of 360 children identified, 256 children met the eligibility criteria and had ascertainable NRPC and primary-outcome data; 164 (64%) were classified as High NRPC and 92 (36%) as Low NRPC. Overall, 37/256 children (14.5%) experienced the primary outcome. The observed event rate was 11.0% (18/164) in the High NRPC group and 20.7% (19/92) in the Low NRPC group, corresponding to a crude OR of 2.11 (95% CI 1.04–4.27; *p* = 0.037) for Low versus High NRPC. In the primary adjusted model, the association was attenuated and imprecise (adjusted OR 1.70, 95% CI 0.80–3.57; *p* = 0.165). Firth penalized regression yielded OR 1.67 (95% CI 0.80–3.49; *p* = 0.168), while separate mixed-effects models yielded OR 1.65 (95% CI 0.76–3.58; *p* = 0.205) for nurse clustering and OR 1.68 (95% CI 0.77–3.66; *p* = 0.190) for surgeon clustering. Per 1-point increase in the NRPC score, the adjusted OR was 0.70 (95% CI 0.48–1.02; *p* = 0.061).

**Conclusion:**

Higher NRPC was associated with lower unadjusted rates of 30-day wound- and pin-site–related adverse events, but the association was attenuated and statistically imprecise after temporal and clinical adjustment. Similar directional estimates across sensitivity analyses support NRPC as an exploratory marker of broader perioperative process quality rather than as an independently established predictor or causal determinant of wound outcomes.

## 1 Introduction

Supracondylar humerus fractures are among the most common elbow injuries requiring operative treatment in children (1). Displaced fractures are generally treated by reduction and percutaneous Kirschner-wire fixation, with open reduction reserved for selected irreducible or unstable fractures (2, 3). Intraoperative fluoroscopy is routinely used to confirm reduction and guide wire placement (4), making radiation-protection practice a routine component of the operative environment.

Radiation-protection frameworks emphasise minimising occupational exposure through time, distance, shielding, training, and procedural controls (5, 6). Orthopaedic literature has focused mainly on surgeons and radiographers, while operating-room nurses also contribute to protective-equipment preparation, procedural coordination, protocol adherence, and safety documentation. These activities may reflect broader attributes of perioperative process organisation as well as radiation-safety practice (7, 8). NRPC is therefore considered here as a pragmatic process-quality marker rather than a factor expected to directly modify wound healing.

Early wound- and pin-site complications remain clinically relevant after percutaneous fixation of supracondylar humerus fractures in children (9, 10). Although many are mild, they may lead to unplanned visits, readmission, or additional intervention (11, 12). Their occurrence is multifactorial and may be influenced by fracture characteristics, patient factors, operative complexity, perioperative antibiotic management, aseptic technique, and wound or pin-site care (13, 14). Because several of these determinants are embedded in team-level perioperative processes, any association between NRPC and wound-related outcomes would more plausibly reflect correlated care processes than a direct biological effect of radiation-protection practice (15).

Evidence specifically linking case-level nursing radiation-protection compliance with postoperative wound- and pin-site outcomes is limited. Radiation protection and surgical-site infection prevention are generally addressed as separate quality domains (16), and routinely recorded nursing process measures have seldom been evaluated in this paediatric orthopaedic setting. Such observational associations cannot disentangle NRPC from correlated nursing, team-level, temporal, or institutional practices.

We therefore conducted a single-centre retrospective observational cohort study to examine the association between case-level NRPC and 30-day wound- and pin-site–related adverse events after fluoroscopy-assisted fixation of supracondylar humerus fractures in children (17). The primary analysis compared Low versus High NRPC with adjustment for prespecified temporal and clinical factors, supported by penalized, ordinal-score, and provider-clustered analyses. NRPC was evaluated as an exploratory marker of broader perioperative process quality rather than as an established causal or predictive factor (18).

## 2 Methods

### 2.1 Study design and setting

This study was designed as a single-centre, retrospective observational cohort conducted at The Second Affiliated Hospital of Wenzhou Medical University in China with an established paediatric orthopaedic and trauma service. Consecutive children who underwent operative treatment for supracondylar humerus fractures between 1 January 2018 and 31 December 2023 were identified through the hospital's electronic medical record (EMR) and operating room information system (ORIS). All data used in the analysis were derived from routine clinical care, including perioperative medical and nursing documentation and follow-up records; no additional procedures, investigations, or protocol-driven modifications of care were introduced for research purposes.

The study protocol was approved by the institutional Ethics Committee of the Second Affiliated Hospital and Yuying Children's Hospital of Wenzhou Medical University (Approval No. 2024079), which granted a waiver of written informed consent because of the retrospective design, use of existing clinical records, and minimal risk to participants. Patient-level data were de-identified before analysis. The study was conducted in accordance with the Declaration of Helsinki and applicable institutional requirements for the use and protection of clinical data. Reporting followed the STROBE recommendations for observational cohort studies, insofar as permitted by the available data.

### 2.2 Participants and perioperative management

#### 2.2.1 Participants and eligibility

Children were eligible if they were aged <14 years, had a radiologically confirmed supracondylar humerus fracture, and underwent operative fixation with intraoperative C-arm fluoroscopy at the study hospital between 1 January 2018 and 31 December 2023. The age threshold was prespecified because it corresponded to the institutional paediatric orthopaedic care pathway used during the study period; children aged 14–17 years were managed outside this pathway and could therefore have differed in perioperative staffing and nursing workflow. Potentially eligible children were identified from the electronic medical record and operating-room information system using diagnostic and procedure records and were subsequently verified by review of operative and nursing documentation.

Children were excluded if they were aged ≥14 years, were managed non-operatively, underwent definitive fracture fixation at another institution, underwent fixation without intraoperative C-arm fluoroscopy, had a pre-existing local infection at or near the operative site, or lacked the information required to ascertain NRPC exposure or the 30-day primary outcome. Children meeting all eligibility criteria and none of the exclusion criteria comprised the analytic cohort.

#### 2.2.2 Perioperative management

Perioperative care followed the institutional clinical pathway for children with supracondylar humerus fractures. All procedures were performed under general anaesthesia with routine paediatric monitoring. In accordance with local surgical-site infection prophylaxis practice, intravenous prophylactic antibiotics were generally administered before skin incision unless contraindicated. Decisions regarding postoperative antibiotic continuation and duration were made by the treating team according to fracture characteristics, soft-tissue condition, operative findings, and the clinical course.

The operative strategy primarily consisted of closed reduction and percutaneous Kirschner-wire fixation, with open reduction reserved for fractures that could not be satisfactorily reduced by closed methods. C-arm fluoroscopy was routinely used intraoperatively to guide reduction, confirm alignment, and verify final Kirschner-wire placement. Postoperatively, the affected limb was immobilised with a cast or splint according to departmental practice. Wound- and pin-site–related complications were ascertained from inpatient records and subsequent outpatient or emergency-department encounters within 30 days after surgery.

### 2.3 Exposure: nursing radiation-protection compliance (NRPC)

The primary exposure was case-level nursing radiation-protection compliance (NRPC), defined in relation to the circulating nurse with primary responsibility for intraoperative nursing care during the index procedure. The NRPC construct was developed as a pragmatic process indicator using information routinely available in the institutional staff-training, nursing quality-management, and perioperative documentation systems. It was not intended as a validated psychometric scale or as a measure of radiation dose.

Three binary components were selected because they represented complementary stages of compliance that could be objectively retrieved from existing records: (1) recent competency preparation, represented by successful completion of formal institutional radiation-protection training and assessment within the preceding 12 months; (2) longitudinal quality-control performance, represented by the absence of radiation-protection–related quality-control deductions during the preceding 12 months; and (3) index-procedure implementation/documentation, represented by explicit documentation of radiation-protection measures during the index operation. Thus, the three components were intended to capture complementary domains of preparation, sustained compliance, and procedure-specific implementation rather than three interchangeable measures of the same behaviour.

For the training component, one point was assigned when the responsible nurse had completed and passed the institutional radiation-protection course within the previous 12 months and zero otherwise. For the quality-control component, one point was assigned when no radiation-protection–related deduction had been recorded for that nurse during the previous 12 months and zero otherwise. For the case-level component, one point was assigned when the surgical safety checklist and/or intraoperative nursing record explicitly documented implementation of appropriate radiation-protection measures during C-arm fluoroscopy and zero otherwise.

The three components were assigned equal weight and summed to produce an NRPC score ranging from 0 to 3. Equal weighting was selected for transparency because no externally validated weighting scheme or prior evidence supported differential weighting of the three domains.

The primary exposure was the prespecified binary classification of Low NRPC (0–1 points) versus High NRPC (2–3 points). Because the institution-derived score has not undergone external validation, the full 0–3 score and the three individual components were examined only in supportive sensitivity or exploratory analyses (Supplementary Table S1). No data-derived weighting or outcome-optimised alternative cut-off was constructed.

All NRPC variables were abstracted using a standardised coding manual. Two trained investigators cross-checked the relevant source records, and discrepancies were resolved by consensus after review of the original documentation. NRPC was interpreted throughout the study as a proxy marker of perioperative nursing process quality rather than as a causal exposure expected to directly modify wound biology.

### 2.4 Outcomes and data collection

#### 2.4.1 Outcomes

The primary outcome was a 30-day composite of wound- and pin-site–related adverse events after the index operation. Events were included if, within 30 days of surgery, new or worsening local signs at the surgical incision or pin sites—such as erythema, swelling, warmth, serous or purulent discharge, or wound dehiscence—were documented in inpatient, outpatient, or emergency department records and judged by the treating team to be related to the operative wound or percutaneous pin tracts. The day of surgery was defined as day 0.

Prespecified secondary outcomes were: (1) events meeting the study definition for surgical site infection (SSI), defined by compatible local findings together with clinician diagnosis and initiation of targeted antimicrobial therapy and/or local intervention; (2) unplanned outpatient visits primarily attributed to wound- or pin-site problems; (3) unplanned hospital readmissions for such problems; and (4) reoperation or surgical debridement for a wound- or pin-site–related complication. For descriptive purposes, primary-outcome events were additionally classified into three mutually exclusive anatomical categories: incision only, pin site only, or both incision and pin site. All outcomes were ascertained from routine clinical documentation during the index admission and subsequent orthopaedic outpatient or emergency-department encounters recorded within 30 days after surgery.

#### 2.4.2 Covariates and data sources

Covariates were selected *a priori* based on clinical plausibility and prior literature on postoperative wound complications. Demographic variables included age (continuous and <5 vs. ≥5 years), sex, and body mass index. Fracture- and injury-related characteristics comprised Gartland classification (type II vs. type III/IV) and open versus closed fracture status. Pre-existing comorbidities were recorded for congenital heart disease and chronic liver or kidney disease. Preoperative physiological and laboratory measures included admission vital signs and routine haematologic, inflammatory, and renal/hepatic function indices where available. Operative variables included surgical technique (closed reduction with percutaneous Kirschner-wire fixation vs. open reduction), operative duration (continuous and >60 vs. ≤60 min), emergency versus elective surgery, and seniority of the primary surgeon. Infection-related management variables captured administration of preoperative prophylactic antibiotics (yes vs. no) and duration of postoperative antibiotic therapy (>24 h vs. ≤24 h or none).

All exposure, outcome, and covariate data were abstracted from the electronic medical record, operating room information system, and perioperative nursing documentation using a standardised electronic case report form. De-identified identifiers for the circulating nurse with primary intraoperative responsibility and the primary surgeon were retained solely to characterise repeated case contributions and to define provider-level clusters for sensitivity analyses. Two trained investigators cross-checked key variables, and any discrepancies were resolved by consensus after review of the original source records.

### 2.5 Statistical analysis

Children with complete information on NRPC exposure and the 30-day primary outcome comprised the analytic cohort; children lacking either variable were excluded, and no missing values were imputed. Continuous variables were summarised as mean (standard deviation) or median (interquartile range), as appropriate, and categorical variables as counts and percentages. Baseline characteristics were compared between High and Low NRPC groups using independent-samples t tests or Mann–Whitney U tests for continuous variables and *χ*^2^ or Fisher's exact tests for categorical variables, as appropriate.

Observed primary-outcome rates were first compared between Low and High NRPC groups using a 2 × 2 contingency table and *χ*^2^ testing, and crude odds ratios (ORs) with 95% confidence intervals (CIs) were estimated using univariable logistic regression. The primary inferential analysis was a parsimonious multivariable logistic regression model with the 30-day composite outcome as the dependent variable and Low versus High NRPC as the exposure of interest. The prespecified adjustment variables were calendar year of surgery, Gartland classification (type III/IV vs. type II), operative duration (>60 vs. ≤60 min), and emergency versus elective surgery. These variables were selected to address secular trends, fracture severity, operative complexity, and surgical urgency rather than on the basis of univariable statistical significance. Calendar year was modelled continuously. Adjusted ORs with 95% CIs were reported. Other measured characteristics, including age, sex, open-fracture status, surgical approach, surgeon seniority, and perioperative antibiotic use, were retained for descriptive analyses but were not simultaneously entered into the primary model.

Sensitivity analyses used Firth penalized logistic regression with the same covariate set as the primary model and separate mixed-effects logistic regression models incorporating a random intercept for the circulating nurse or the primary surgeon. The same fixed-effect covariates were retained in both mixed-effects models, and provider-level intraclass correlation coefficients (ICCs) were estimated. Nurse and surgeon clustering were evaluated separately rather than with a crossed random-effects structure to limit model complexity. The robustness of the exposure definition was additionally examined by modelling the total NRPC score as an ordinal variable from 0 to 3, with the OR expressed per 1-point increase. A Cochran–Armitage trend test was used to describe the unadjusted trend in event proportions across NRPC scores, and the three individual NRPC components were examined as correlated exploratory analyses (Supplementary Table S1). Provider structure and provider-clustered models were additionally evaluated as sensitivity analyses (Supplementary Table S3). No subgroup or exposure-by-covariate interaction analyses were prespecified or performed. Given the retrospective observational design and exploratory nature of the NRPC construct, interpretation emphasised the magnitude, direction, and precision of effect estimates and their 95% CIs rather than statistical significance alone. All tests were two-sided. Analyses were performed using IBM SPSS Statistics, version 32.0.0 (IBM Corp., Armonk, NY, USA) and R, version 4.6.1 (R Foundation for Statistical Computing, Vienna, Austria); Firth penalized logistic regression was implemented in R.

## 3 Results

### 3.1 Patient selection and baseline characteristics

During the study period, 360 children with supracondylar humerus fractures were identified from the electronic medical record and operating-room information systems. Eighteen children aged ≥14 years, 54 who were managed non-operatively, 10 who underwent fixation without intraoperative C-arm fluoroscopy, 8 with a pre-existing local infection at or near the operative site, and 14 with missing key NRPC exposure or 30-day primary-outcome data were excluded. The final analytic cohort therefore comprised 256 children (Figure 1). All included children had ascertainable NRPC exposure and primary-outcome data. According to the prespecified classification, 164 procedures (64%) were classified as High NRPC and 92 (36%) as Low NRPC (Table 1).

**Figure 1:**
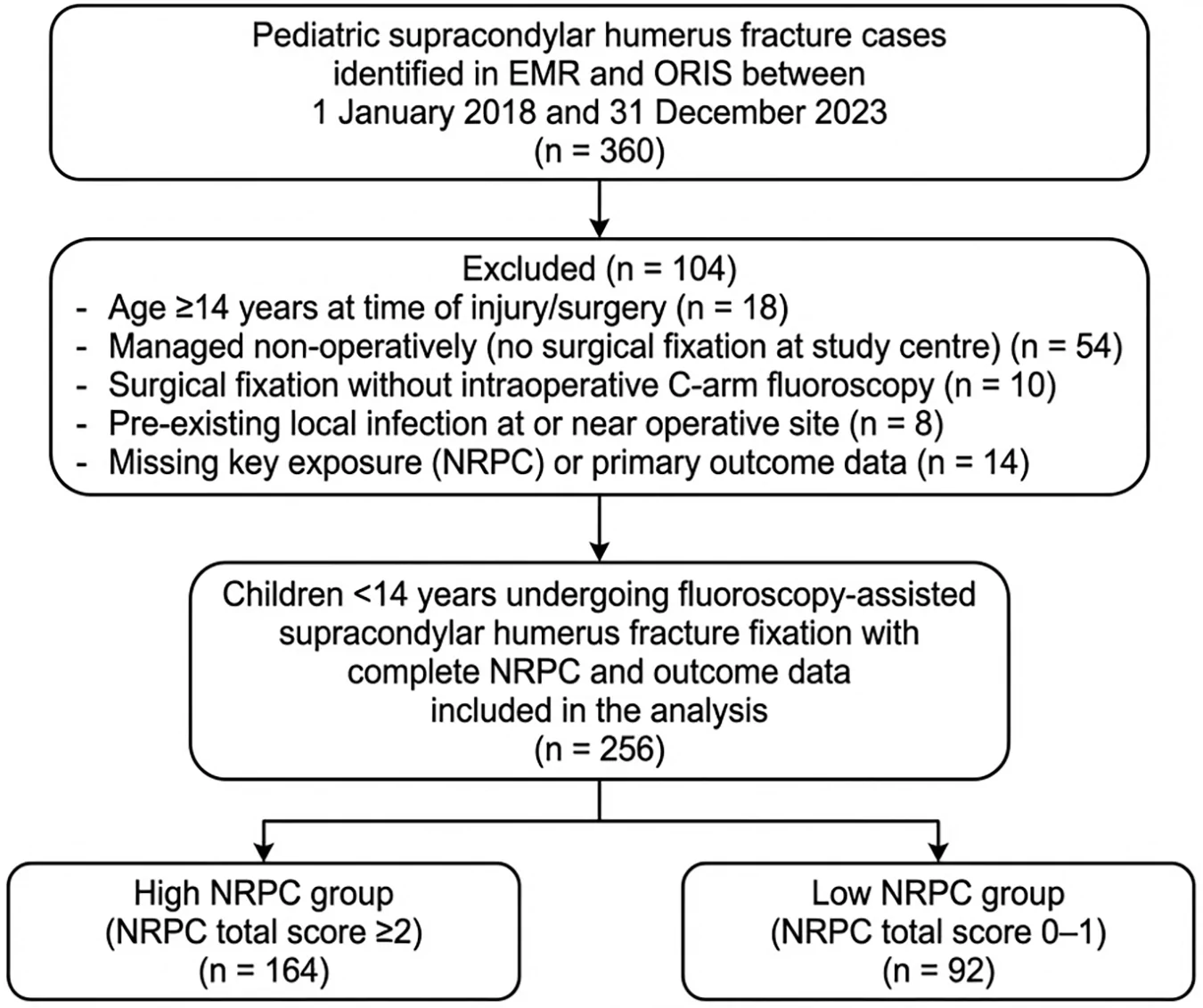
Flow of children from screening to the final retrospective cohort and classification by nursing radiation-protection compliance (NRPC). Flowchart of cohort selection and classification by nursing radiation-protection compliance (NRPC) group. A total of 360 children with supracondylar humerus fractures were identified in the electronic medical record (EMR) and operating room information system (ORIS) between 1 January 2018 and 31 December 2023. After exclusion of children aged ≥14 years at the time of injury or surgery, those managed non-operatively, those undergoing surgical fixation without intraoperative C-arm fluoroscopy, those with pre-existing local infection at or near the operative site, and those with missing key exposure (NRPC) or primary outcome data, 256 children were included in the final analysis and classified into a High NRPC group (NRPC total score ≥2; *n* = 164) and a Low NRPC group (NRPC total score 0–1; *n* = 92).

**Table 1:** Baseline demographic, clinical, fracture, and perioperative characteristics of children undergoing fluoroscopy-assisted supracondylar humerus fracture fixation, stratified by nursing radiation-protection compliance group.

Variable	High NRPC group (*n* = 164)	Low NRPC group (*n* = 92)	Statistical test (test statistic)	p value
Demographics				
Age, years, median (IQR)	6.3 (4.8–8.1)	6.5 (5.1–8.4)	Mann–Whitney U (Z = −0.50)	0.620
Age <5 years, n/N (%)	60/164 (37%)	35/92 (38%)	*χ*^2^ = 0.10	0.752
Male sex, n/N (%)	104/164 (63%)	60/92 (65%)	χ^2^ = 0.08	0.777
Body mass index, kg/m^2^, mean (SD)	17.2 (2.1)	17.0 (2.0)	t = 0.55	0.582
Fracture characteristics				
Gartland type III/IV, n/N (%)	88/164 (54%)	52/92 (57%)	χ^2^ = 0.18	0.674
Open fracture, n/N (%)	3/164 (2%)	3/92 (3%)	χ^2^ = 0.16	0.690
Left side involved, n/N (%)	98/164 (60%)	52/92 (57%)	χ^2^ = 0.22	0.641
Comorbidities				
Congenital heart disease, n/N (%)	3/164 (2%)	1/92 (1%)	χ^2^ = 0.03	0.859
Chronic liver or kidney disease, n/N (%)	1/164 (1%)	0/92 (0%)	Fisher's exact test (not reported)	0.999
Admission vital signs				
Systolic blood pressure, mmHg, mean (SD)	102.4 (9.1)	103.8 (9.8)	t = −0.81	0.417
Heart rate, beats/min, mean (SD)	105.2 (11.8)	107.3 (11.1)	t = −1.06	0.291
Laboratory values—haematology and inflammation				
Haemoglobin, g/L, mean (SD)	118.6 (10.2)	117.3 (11.0)	t = 0.71	0.475
White blood cell count, ×10⁹/L, median (IQR)	9.8 (7.5–12.3)	10.1 (7.8–12.8)	Mann–Whitney U (Z = −0.57)	0.568
C-reactive protein, mg/L, median (IQR)	4.2 (1.5–9.8)	4.8 (1.7–10.6)	Mann–Whitney U (Z = −0.52)	0.601
Laboratory values—renal function				
Serum creatinine, μmol/L, median (IQR)	32.0 (26.0–38.0)	33.0 (27.0–39.0)	Mann–Whitney U (Z = −0.35)	0.726
eGFR, mL·min⁻^1^·1.73 m⁻^2^, mean (SD)	140.5 (18.2)	138.1 (19.0)	t = 0.90	0.370
Laboratory values—liver function				
ALT, U/L, median (IQR)	18.0 (13.0–26.0)	19.0 (14.0–28.0)	Mann–Whitney U (Z = −0.45)	0.652
AST, U/L, median (IQR)	24.0 (19.0–32.0)	25.0 (20.0–34.0)	Mann–Whitney U (Z = −0.54)	0.590
Surgical characteristics				
CRPP as surgical approach, n/N (%)	132/164 (80%)	72/92 (78%)	χ^2^ = 0.16	0.689
Duration of surgery, min, median (IQR)	55.0 (45.0–70.0)	58.0 (47.0–75.0)	Mann–Whitney U (Z = −1.01)	0.313
Duration of surgery >60 min, n/N (%)	54/164 (33%)	35/92 (38%)	χ^2^ = 0.67	0.413
Emergency surgery, n/N (%)	46/164 (28%)	32/92 (35%)	χ^2^ = 1.18	0.277
Senior surgeon as primary operator^a^, n/N (%)	112/164 (68%)	56/92 (61%)	χ^2^ = 1.43	0.232
Perioperative antibiotic use				
Preoperative prophylactic antibiotics, n/N (%)	154/164 (94%)	86/92 (94%)	χ^2^ = 0.01	0.914
Postoperative antibiotics >24 h, n/N (%)	38/164 (23%)	26/92 (28%)	χ^2^ = 0.74	0.389

a Senior surgeon defined as chief, associate chief, or senior attending surgeon.

ALT, alanine aminotransferase; AST, aspartate aminotransferase; CRPP, closed reduction and percutaneous pinning; eGFR, estimated glomerular filtration rate; IQR, interquartile range; NRPC, nursing radiation-protection compliance; SD, standard deviation. Data are mean (SD), median (IQR), or n/N (%) as indicated.

Baseline demographic, clinical, and perioperative characteristics were broadly comparable between the two NRPC strata (Table 1). The median age was 6.3 years (IQR 4.8–8.1) in the High NRPC group and 6.5 years (5.1–8.4) in the Low NRPC group, with children younger than 5 years accounting for 37% (60/164) and 38% (35/92), respectively. Boys represented 63% (104/164) of the High NRPC group and 65% (60/92) of the Low NRPC group, and mean body mass index was 17.2 and 17.0 kg/m^2^, respectively. Approximately half of fractures in each group were Gartland type III/IV (54% [88/164] vs. 57% [52/92]), and open injuries were uncommon (2% [3/164] vs. 3% [3/92]). Comorbid congenital heart disease and chronic liver or kidney disease were infrequent overall. Admission vital signs, haematologic and inflammatory markers, and renal and hepatic function tests showed no meaningful between-group differences. Most children underwent closed reduction and percutaneous pinning (80% [132/164] vs. 78% [72/92]), with median operative durations of 55 min (IQR 45–70) and 58 min (47–75); procedures lasted >60 min in 33% (54/164) and 38% (35/92) of the High and Low NRPC groups, respectively. Emergency surgery accounted for 28% (46/164) versus 35% (32/92), and senior surgeons served as the primary operator in 68% (112/164) versus 61% (56/92). Preoperative prophylactic antibiotics were administered in 94% of procedures in both groups (154/164 vs. 86/92), and postoperative antibiotics were continued for more than 24 h in 23% (38/164) and 28% (26/92), respectively. No large between-group differences were apparent for the measured baseline characteristics; however, these unadjusted comparisons do not exclude residual differences in case complexity, temporal practice patterns, or other unmeasured factors (Table 1).

### 3.2 Nursing radiation-protection compliance and score distribution

Among the 256 procedures, 190 (74%) were performed by nurses who had completed formal radiation-protection training and passed assessment within the previous 12 months, including 150/164 (91%) procedures in the High NRPC group and 40/92 (44%) in the Low NRPC group (*p* < 0.001). Similarly, 160 procedures (63%) were performed by nurses with no radiation-protection–related quality-control deductions in the preceding 12 months, including 140/164 (85%) in the High NRPC group and 20/92 (22%) in the Low NRPC group (*p* < 0.001). Case-level documentation indicating that appropriate radiation-protection measures were implemented during surgery was present in 110 procedures (43%), including 90/164 (55%) in the High NRPC group and 20/92 (22%) in the Low NRPC group (*p* < 0.001). As expected from the score definition, the three component criteria were more frequently present among procedures classified as High NRPC than among those classified as Low NRPC (Table 2).

**Table 2:** Components and distribution of the nursing radiation-protection compliance (NRPC) score and observed 30-day wound- and pin-site–related adverse-event rates.

Panel A. Components of NRPC by compliance group					
Component of NRPC^a^	Overall (*n* = 256)	High NRPC group^b^ (*n* = 164)	Low NRPC group^c^ (*n* = 92)	Statistical test (test statistic)	p value
Completed radiation-protection training and passed assessment within previous 12 months	190/256 (74%)	150/164 (91%)	40/92 (44%)	χ^2^ = 70.9	<0.001
No radiation-protection–related quality-control deductions in previous 12 months	160/256 (63%)	140/164 (85%)	20/92 (22%)	χ^2^ = 101.8	<0.001
Case-level documentation of radiation-protection measures implemented during surgery^d^	110/256 (43%)	90/164 (55%)	20/92 (22%)	χ^2^ = 26.4	<0.001

Panel B. NRPC total score (0–3) and 30 day wound and pin site–related adverse events			
NRPC total score (0–3)	Children, n/N (%)	Children with 30-day wound-/pin-site–related adverse events, n/N (%)	Event rate (%)
0	30/256 (12%)	9/30 (30.0%)	30.0
1	62/256 (24%)	10/62 (16.1%)	16.1
2	94/256 (37%)	11/94 (11.7%)	11.7
3	70/256 (27%)	7/70 (10.0%)	10.0
Total	**256/256 (100%)**	**37/256 (14.5%)**	**14**.**5**

Data are n/N (%) unless otherwise stated.

a NRPC, nursing radiation-protection compliance, composed of three binary items (training, quality-control record, and case-level documentation) scored 0–1 point each, yielding a total score from 0 to 3.

b High NRPC group defined as NRPC total score ≥2 (*n* = 164).

c Low NRPC group defined as NRPC total score 0–1 (*n* = 92).

d Documentation from the surgical safety checklist and/or intraoperative nursing record explicitly indicating that appropriate radiation-protection measures were implemented during C-arm fluoroscopy. Because Low and High NRPC groups were defined from the three component items, between-group comparisons of individual components are descriptive and should not be interpreted as independent inferential tests.

Trend test for decreasing event rates across NRPC scores: Cochran–Armitage test for trend, Z = −2.46, *p* = 0.014.

Bold values indicate *p* < 0.05.

The NRPC total score ranged from 0 to 3. Scores of 0, 1, 2, and 3 were observed in 30 (12%), 62 (24%), 94 (37%), and 70 (27%) procedures, respectively (Table 2; Figure 2). The corresponding observed 30-day primary-outcome rates were 30.0% (9/30), 16.1% (10/62), 11.7% (11/94), and 10.0% (7/70). A Cochran–Armitage test indicated a decreasing unadjusted trend in event proportions across increasing NRPC scores (Z = −2.46; *p* = 0.014). Regression analyses using the ordinal score are reported with the other sensitivity analyses in Section 3.4.

**Figure 2:**
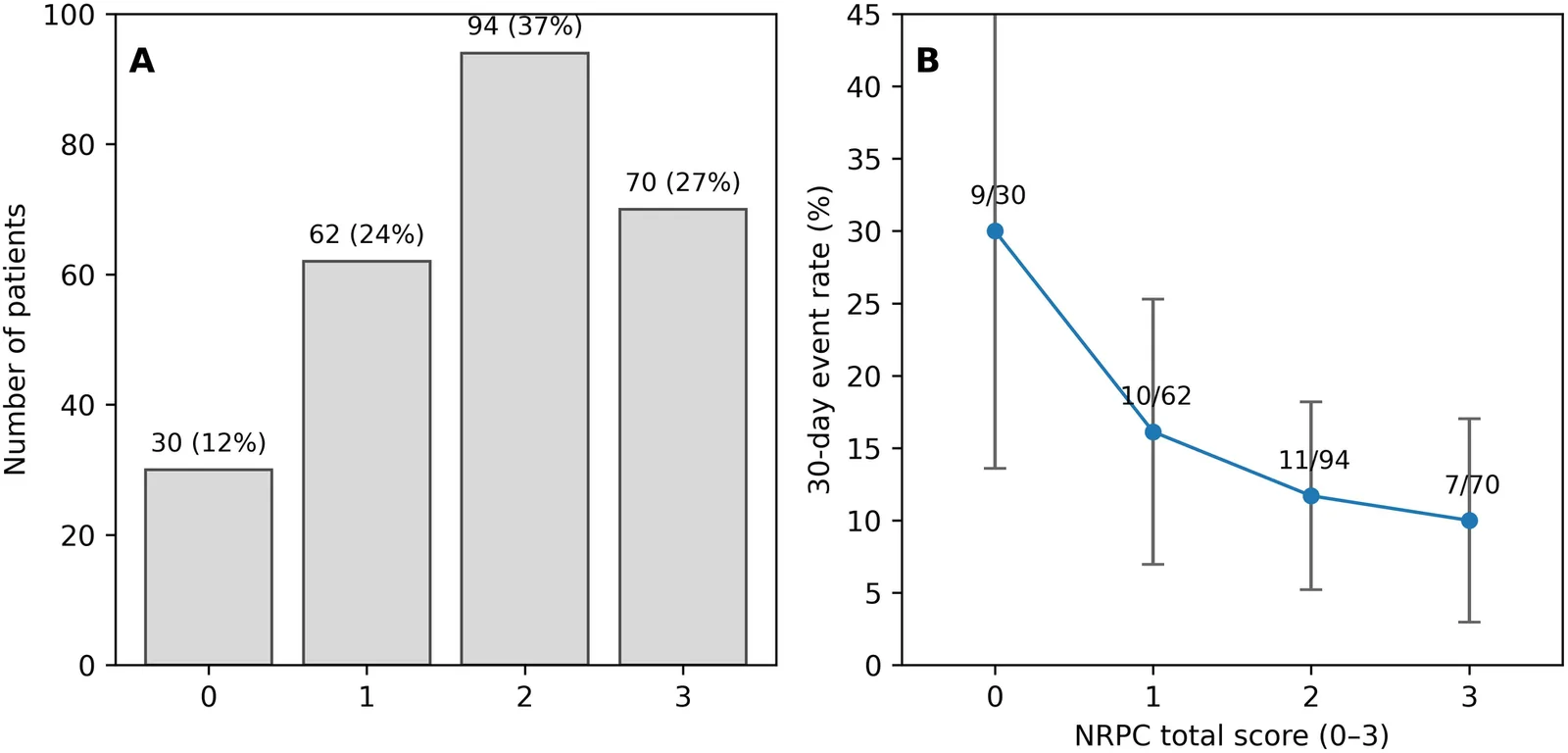
Distribution of nursing radiation-protection compliance (NRPC) scores and observed 30-day wound- and pin-site–related adverse-event rates. **(A)** Distribution of NRPC total scores (0–3) among 256 children undergoing fluoroscopy-assisted supracondylar humerus fracture fixation. Bars show the number and percentage of children within each NRPC score category. **(B)** Crude 30-day wound- and pin-site–related adverse event rates according to NRPC total score. Points represent observed event rates with 95% confidence intervals, with labels indicating the number of events and total children in each NRPC score category (events/total).

NRPC also varied across the study period. The proportion of procedures classified as High NRPC increased from 44.7% in 2018 to 80.9% in 2023, while the observed 30-day adverse-event rate declined from 21.1% to 8.5% over the same period (Supplementary Table S2). Because these parallel temporal patterns could reflect secular changes in nursing governance, documentation, infection-prevention practice, or other institutional processes, calendar year was included in the adjusted analyses.

### 3.3 Thirty-day wound- and pin-site–related outcomes by NRPC group

Within 30 days of surgery, 37 of 256 children (14.5%) experienced at least one wound- or pin-site–related adverse event, corresponding to the primary composite outcome (Table 3). The observed event rate was 11.0% in the High NRPC group (18/164) and 20.7% in the Low NRPC group (19/92; *χ*^2^ = 4.46; *p* = 0.035) (Table 3; Figure 3). Consequently, 219 of 256 children (85.5%) had no wound- or pin-site–related adverse event within 30 days, comprising 146/164 (89.0%) in the High NRPC group and 73/92 (79.3%) in the Low NRPC group. Events meeting the prespecified study definition for SSI were less frequent, occurring in 12/256 children (4.7%): 5/164 (3.0%) in the High NRPC group and 7/92 (7.6%) in the Low NRPC group (*χ*^2^ = 2.74; *p* = 0.098). Wound- or pin-site–related complications requiring an unplanned outpatient visit were observed in 25 children (9.8%), including 12/164 (7.3%) in the High NRPC group and 13/92 (14.1%) in the Low NRPC group. Unplanned readmissions attributable to wound- or pin-site problems occurred in 8/256 children (3.1%; 3/164 [1.8%] vs. 5/92 [5.4%]), and 4/256 (1.6%; 1/164 [0.6%] vs. 3/92 [3.3%]) required reoperation or surgical debridement (Table 3). These utilisation endpoints were not mutually exclusive, and individual children could contribute to more than one category.

**Table 3:** Thirty-day wound- and pin-site–related adverse events, healthcare utilisation, and anatomical distribution by nursing radiation-protection compliance (NRPC) group.

Panel A. Burden and healthcare utilisation related to 30 day wound and pin site–related adverse events					
Outcome within 30 days	Overall (*N* = 256)	High NRPC group^a^ (*N* = 164)	Low NRPC group^b^ (*N* = 92)	Statistical test (test statistic)	p value
Primary composite outcome: any wound-/pin-site–related adverse event	37/256 (14.5%)	18/164 (11.0%)	19/92 (20.7%)	χ^2^ = 4.46	0.035
No wound-/pin-site–related adverse event	219/256 (85.5%)	146/164 (89.0%)	73/92 (79.3%)	–	–
Events meeting study-defined surgical site infection (SSI) criteria^c^	12/256 (4.7%)	5/164 (3.0%)	7/92 (7.6%)	χ^2^ = 2.74	0.098
Wound-/pin-site–related complications requiring unplanned outpatient visit^d^	25/256 (9.8%)	12/164 (7.3%)	13/92 (14.1%)	–	–
Wound-/pin-site–related unplanned readmission	8/256 (3.1%)	3/164 (1.8%)	5/92 (5.4%)	–	–
Wound-/pin-site–related reoperation or surgical debridement	4/256 (1.6%)	1/164 (0.6%)	3/92 (3.3%)	–	–

Panel B. Anatomical distribution of 30 day wound and pin site–related adverse events‖			
Anatomical pattern of adverse event	Overall (*N* = 256)	High NRPC group (*N* = 164)	Low NRPC group (*N* = 92)
Pin-site complications only	24/256 (9.4%)	11/164 (6.7%)	13/92 (14.1%)
Incision complications only	9/256 (3.5%)	5/164 (3.0%)	4/92 (4.3%)
Both incision and pin-site complications	4/256 (1.6%)	2/164 (1.2%)	2/92 (2.2%)

Data are n/N (%) unless otherwise stated.

a High NRPC group defined as NRPC total score ≥2.

b Low NRPC group defined as NRPC total score 0–1.

c SSI denotes events meeting the prespecified study definition: compatible local wound or pin-site findings together with clinician diagnosis and initiation of targeted antimicrobial therapy and/or local intervention.

d Categories in Panel A are not mutually exclusive; individual children may contribute to more than one category (for example, SSI with both outpatient visit and readmission).

Categories in Panel B are mutually exclusive; each child with at least one 30-day wound- or pin-site–related adverse event is counted in a single anatomical pattern category (total 37 children, corresponding to the primary composite outcome in Panel A).

NRPC, nursing radiation-protection compliance; SSI, surgical site infection.

**Figure 3:**
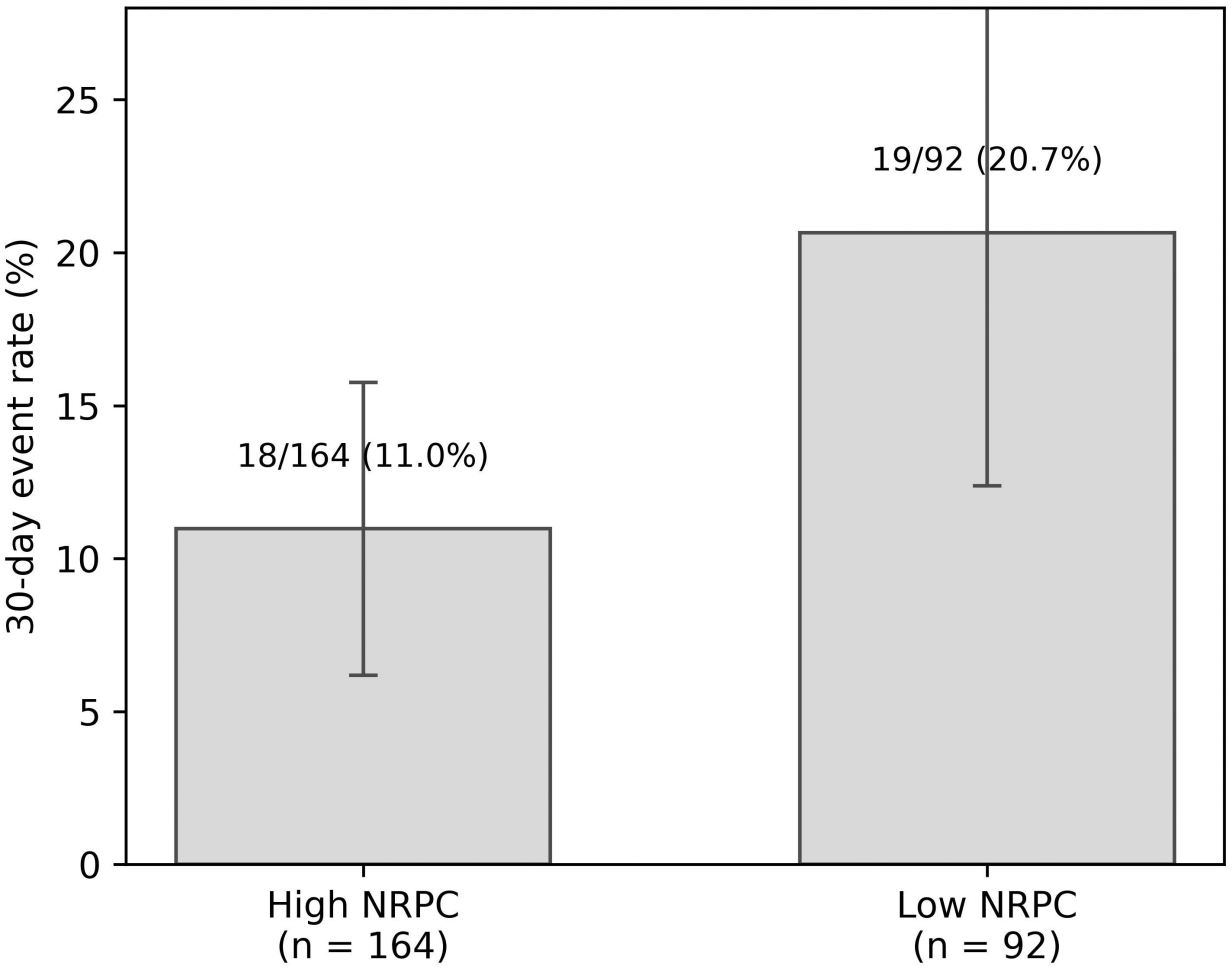
Observed 30-day wound- and pin-site–related adverse-event rates by nursing radiation-protection compliance (NRPC) group. Bars show the unadjusted 30-day event rates in the High NRPC group (NRPC score 2–3; 18/164, 11.0%) and Low NRPC group (NRPC score 0–1; 19/92, 20.7%). Vertical lines indicate 95% confidence intervals for the observed proportions. The corresponding crude odds ratio for Low versus High NRPC was 2.11 (95% CI 1.04–4.27). Corresponding adjusted estimates are reported separately (Figure 4).

When outcomes were classified by anatomical pattern, most 30-day adverse events involved the pin sites alone (24/256 [9.4%]), with fewer events confined to the surgical incision (9/256 [3.5%]) or affecting both incision and pin sites (4/256 [1.6%]) (Table 3). Among children in the High NRPC group, pin-site–only complications occurred in 11/164 (6.7%), incision-only complications in 5/164 (3.0%), and combined incision and pin-site complications in 2/164 (1.2%). The corresponding proportions in the Low NRPC group were 13/92 (14.1%), 4/92 (4.3%), and 2/92 (2.2%), respectively. These mutually exclusive anatomical categories account for all 37 children with at least one wound- or pin-site–related adverse event and mirror the distribution of the primary composite outcome across NRPC groups (Table 3).

### 3.4 Crude, adjusted, and sensitivity analyses of NRPC and 30-day adverse events

In univariable logistic regression, Low NRPC was associated with higher crude odds of the 30-day composite outcome than High NRPC (OR 2.11, 95% CI 1.04–4.27; *p* = 0.037) (Table 4; Figure 4).

**Table 4:** Crude, multivariable-adjusted, and firth-penalized associations with 30-day wound- and pin-site–related adverse events.

Predictor	Crude OR (95% CI)	P	Adjusted OR (95% CI)	P	Firth-adjusted OR (95% CI)	P
Low vs. High NRPC	2.11 (1.04–4.27)	0.037	**1.70** **(0.80–3.57)**	**0**.**165**	**1.67** (**0.80–3.49)**	**0**.**168**
Calendar year, per 1-year later	**0.75** (**0.60–0.93)**	**0**.**008**	**0.85** (**0.68–1.07)**	**0**.**161**	**0.86** (**0.69–1.07)**	**0**.**170**
Gartland III/IV vs. II	1.88 (0.90–3.94)	0.092	**1.93** (**0.90–4.10)**	**0**.**089**	**1.86** (**0.88–3.96)**	**0**.**106**
Duration >60 vs. ≤60 min	1.97 (0.98–3.99)	0.058	**1.94** (**0.94–4.01)**	**0**.**072**	**1.91** (**0.94–3.90)**	**0**.**075**
Emergency vs. elective	1.69 (0.82–3.46)	0.153	**1.60** (**0.76–3.37)**	**0**.**218**	**1.59** (**0.77–3.31)**	**0**.**212**

NRPC, nursing radiation-protection compliance; OR, odds ratio; CI, confidence interval.

Adjusted estimates were obtained from a parsimonious multivariable logistic regression including NRPC group, calendar year of surgery, Gartland classification, operative duration, and emergency surgical status. Calendar year was modelled continuously. Firth penalized logistic regression used the same covariate set. Because only 37 primary outcome events occurred, adjusted analyses were intentionally parsimonious and should be considered exploratory. Additional mixed-effects sensitivity analyses accounted separately for repeated procedures contributed by the same circulating nurse and primary surgeon (Supplementary Table S3; Figure 4). The conventional multivariable logistic regression model was the primary inferential model; Firth penalized regression was performed as a sensitivity analysis using the same covariate set.

Bold values indicate *p* < 0.05.

**Figure 4:**
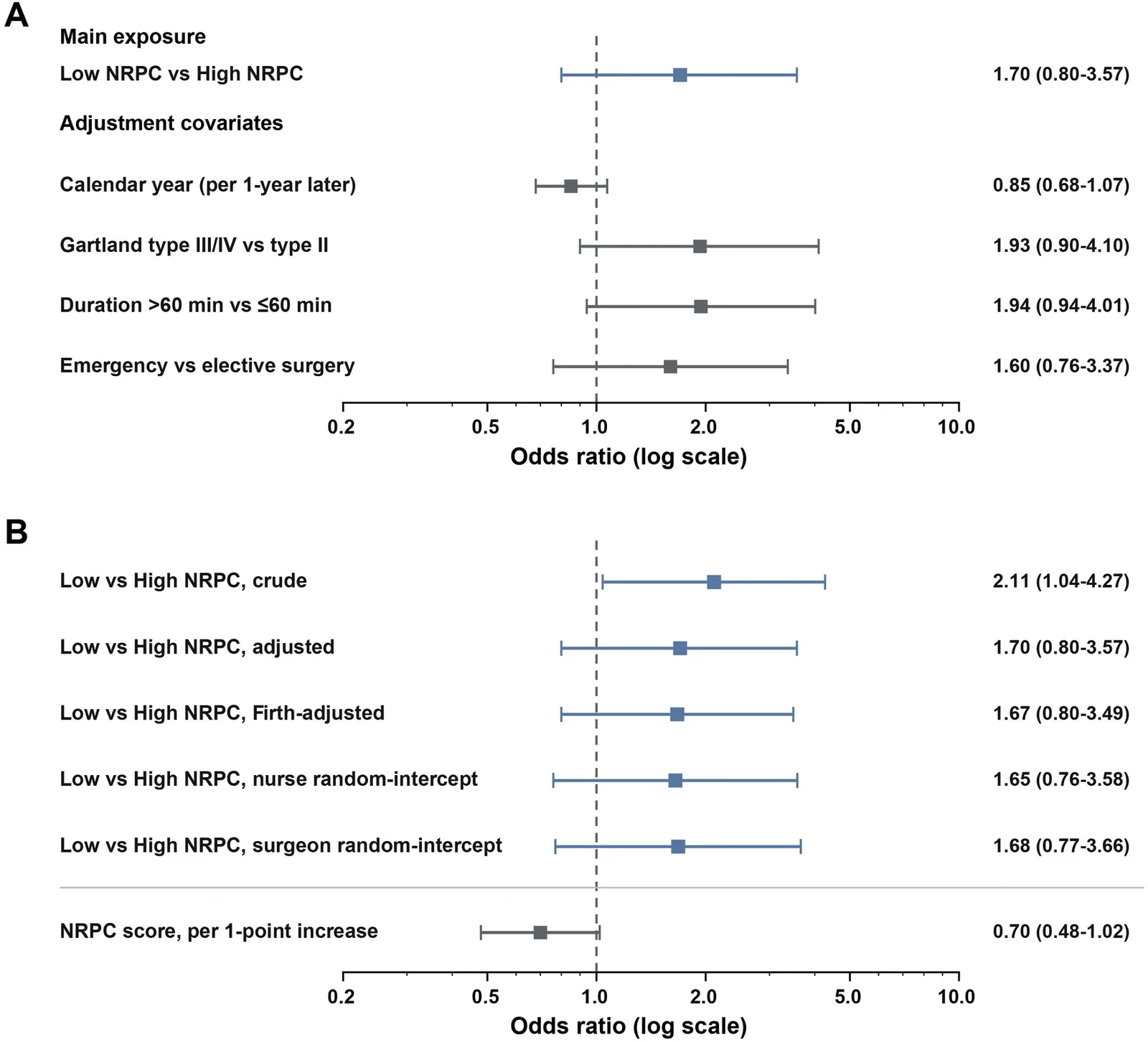
Primary adjusted and sensitivity estimates for the association between nursing radiation-protection compliance (NRPC) and 30-day wound- and pin-site–related adverse events. **(A)** Forest plot of the primary multivariable logistic regression model for Low versus High NRPC, adjusted for calendar year of surgery, Gartland classification, operative duration, and emergency surgical status. **(B)** Sensitivity analyses showing the crude estimate, the primary conventionally adjusted estimate, the Firth-penalized estimate, estimates from separate mixed-effects logistic regression models with random intercepts for the circulating nurse and primary surgeon, and the adjusted association per 1-point increase in the 0–3 NRPC score. Squares denote odds ratios (ORs), horizontal lines denote 95% confidence intervals (CIs), and the dashed vertical line denotes the null value (OR = 1.0). All adjusted provider-clustered models used the same fixed-effect covariate set as the primary model. Adjusted sensitivity estimates were directionally similar but imprecise, with confidence intervals compatible with no independent association.

In the prespecified primary multivariable model adjusting for calendar year, Gartland classification, operative duration, and emergency surgical status, the association was attenuated and imprecisely estimated (adjusted OR 1.70, 95% CI 0.80–3.57; *p* = 0.165). Within the same model, the adjusted ORs were 0.85 (95% CI 0.68–1.07; *p* = 0.161) per successive calendar year, 1.93 (95% CI 0.90–4.10; *p* = 0.089) for Gartland type III/IV versus type II, 1.94 (95% CI 0.94–4.01; *p* = 0.072) for operative duration >60 versus ≤60 min, and 1.60 (95% CI 0.76–3.37; *p* = 0.218) for emergency versus elective surgery.

Firth penalized logistic regression using the same covariates yielded a similar estimate for Low versus High NRPC (OR 1.67, 95% CI 0.80–3.49; *p* = 0.168) (Table 4). The 256 procedures were contributed by 18 circulating nurses and 12 primary surgeons. Circulating nurses contributed a median of 13 procedures (IQR 9–18; range 5–24), and primary surgeons a median of 20.5 procedures (IQR 14–27; range 7–35) (Supplementary Table S3). De-identified provider identifiers were available for all included procedures. In separate mixed-effects sensitivity analyses, the estimate for Low versus High NRPC was OR 1.65 (95% CI 0.76–3.58; *p* = 0.205) with a circulating-nurse random intercept and OR 1.68 (95% CI 0.77–3.66; *p* = 0.190) with a primary-surgeon random intercept. The corresponding ICCs were 0.04 and 0.03, respectively (Figure 4; Supplementary Table S3).

When the full NRPC score was modelled as an ordinal exposure, each 1-point increase was associated with a crude OR of 0.64 (95% CI 0.45–0.92; *p* = 0.015), an adjusted OR of 0.70 (95% CI 0.48–1.02; *p* = 0.061), and a Firth-adjusted OR of 0.70 (95% CI 0.48–1.02; *p* = 0.066). In exploratory component-level analyses, recent radiation-protection training was associated with an adjusted OR of 0.44 (95% CI 0.21–0.94; *p* = 0.034), whereas the estimates for absence of recent quality-control deductions (OR 0.67, 95% CI 0.32–1.39; *p* = 0.282) and case-level documentation of radiation-protection measures (OR 0.79, 95% CI 0.37–1.67; *p* = 0.538) were less precise (Supplementary Table S1). Because these components were correlated by construction and were examined exploratorily, no component was interpreted as an independently established determinant of the outcome.

Across the primary, penalized, provider-clustered, and ordinal-score analyses, point estimates were directionally similar, while adjusted confidence intervals remained compatible with no independent association.

## 4 Discussion

In this retrospective cohort of 256 children undergoing fluoroscopy-assisted fixation of supracondylar humerus fractures, the observed 30-day wound- and pin-site–related adverse-event rate was lower in the High NRPC group than in the Low NRPC group (11.0% vs. 20.7%). However, the association was attenuated in the prespecified primary model after adjustment for calendar year, Gartland classification, operative duration, and emergency surgical status (adjusted OR for Low vs. High NRPC 1.70, 95% CI 0.80–3.57). Firth penalized and provider-clustered sensitivity analyses yielded similar point estimates but remained imprecise, and the association per 1-point increase in the NRPC score was similarly weakened after adjustment (19). Taken together, these findings indicate an exploratory association between NRPC and early wound- and pin-site–related outcomes but do not establish NRPC as an independent predictor or causal determinant.

These findings extend previous work in two directions. First, they add to the literature on short-term wound- and pin-site complications after supracondylar humerus fracture fixation by providing data from a real-world cohort of children using routinely collected clinical and nursing records rather than purpose-built registries (20). The observed pattern, in which most events involved pin sites and relatively few met the prespecified study definition for surgical site infection or required reoperation, is consistent with earlier reports that many postoperative concerns in this setting are mild but clinically relevant (11, 21). Second, our analysis focuses on nursing radiation-protection compliance, which has received far less attention than surgeon or radiographer radiation exposure in orthopaedic theatres (7). By combining training status, quality-control history, and procedure-specific documentation, the NRPC index provides a pragmatic case-level measure that can be derived from existing institutional systems (22).

The observed association should not be interpreted as evidence that radiation-protection practices themselves alter wound biology. A more plausible conceptual interpretation is that NRPC captures aspects of broader perioperative process organisation, including training engagement, protocol adherence, documentation, and other correlated nursing or institutional practices (22). The present data cannot separate NRPC from these correlated processes. Accordingly, the decreasing crude event rates across the 0–3 score are compatible with a process-quality marker but should not be interpreted as a causal dose–response relationship.

Temporal confounding is particularly relevant to interpretation of these findings. The proportion of procedures classified as High NRPC increased during the study period while the observed adverse-event rate decreased, and inclusion of calendar year together with clinical covariates attenuated the NRPC estimate. Secular changes in nursing governance, documentation, infection-prevention practice, or other institutional processes could therefore account for part of the crude association. Higher fracture severity, longer operative duration, and emergency surgery also showed directionally higher estimates, consistent with greater baseline risk in more complex or urgent cases (23). Because only 37 primary-outcome events occurred, the primary model was intentionally parsimonious and the resulting confidence intervals remained wide (24). Similar estimates across conventional, Firth-penalized, and provider-clustered models suggest that the direction of the estimate was not dependent on a single modelling approach, but the data remained compatible with no independent association and should be interpreted as exploratory (25).

From a practical perspective, the NRPC score has the advantage of relying entirely on information already available in training databases, quality-management systems, and routine perioperative documentation. The three components were selected to represent complementary aspects of recent competency preparation, longitudinal quality-control performance, and index-procedure implementation/documentation. Equal weighting was used for transparency and simplicity because no validated weighting scheme was available. The score was therefore intended as a pragmatic formative process index rather than a formally validated measurement scale, and neither the component weights nor the Low/High threshold should be regarded as empirically optimal. Accordingly, alternative weighting strategies should be evaluated prospectively or in independent validation cohorts rather than derived from the present outcome data. Importantly, this institution-derived score has not undergone formal external validation. Although analyses across the full 0–3 range and the individual components provided some evidence that the overall finding was not solely dependent on the binary Low/High categorisation, this internal robustness does not establish the score as a validated performance metric. Its current role should therefore be limited to an exploratory process-quality indicator rather than a tool for routine benchmarking or causal inference.

This study has several limitations. First, it was conducted at a single tertiary hospital, and the findings may not generalise to institutions with different populations of children, staffing structures, perioperative pathways, or nursing quality-control systems (26). The cohort was restricted to children aged <14 years because this age threshold defined the institutional paediatric orthopaedic pathway, and the findings should not be directly extrapolated to older adolescents managed under different workflows. Second, the retrospective design is susceptible to residual confounding and exposure or outcome misclassification despite predefined definitions and standardised data abstraction. In particular, secular changes in nursing governance, documentation, infection-prevention practices, and other institutional processes may have affected both NRPC and postoperative outcomes. Third, 14 potentially eligible children were excluded because key exposure or primary-outcome information was unavailable, introducing the possibility of selection bias. Fourth, only 37 primary-outcome events occurred, limiting the number of covariates that could be included and resulting in imprecise adjusted estimates (27). The primary model therefore did not simultaneously adjust for all measured characteristics, including surgical approach, surgeon seniority, perioperative antibiotic management, open-fracture status, and uncommon comorbidities. Fifth, repeated procedures were contributed by 18 circulating nurses and 12 primary surgeons. Separate random-intercept analyses produced low estimated ICCs and similar NRPC estimates, but they cannot eliminate provider-level confounding or fully represent cross-classified nurse–surgeon team effects. Finally, the NRPC score is an institution-derived process index whose component weighting and Low/High threshold have not undergone external validation.

## 5 Conclusion

In this single-centre retrospective cohort, higher nursing radiation-protection compliance was associated with lower observed rates of 30-day wound- and pin-site–related adverse events after fluoroscopy-assisted fixation of supracondylar humerus fractures in children. The association was attenuated and imprecisely estimated after adjustment for calendar year, fracture severity, operative duration, and emergency surgical status, with directionally similar estimates in penalized and provider-clustered sensitivity analyses. These findings support further investigation of NRPC as an exploratory marker of broader perioperative process quality but do not establish it as an independent predictor, validated quality metric, or causal determinant of wound outcomes. External validation in larger multicentre cohorts is required before the NRPC index can be considered for routine quality assessment.

## Data Availability

The raw data supporting the conclusions of this article will be made available by the authors, without undue reservation.
