# Supplementary material for: Nursing radiation-protection compliance and 30-day wound and pin-site adverse events after supracondylar humerus fracture fixation in children
Source: Front Pediatr. 2026 Oct 15;14:1902962. doi: 10.3389/fped.2026.1902962 (PMC13716741; doi:10.3389/fped.2026.1902962)
Supplement: Supplementary file 1 [file Table1.docx]

**Supplementary Table S1**

**Supplementary Table S1. Sensitivity analyses using alternative representations of nursing radiation-protection compliance**

| NRPC exposure definition | Events / total | Crude OR (95% CI) | P value | Adjusted OR (95% CI)* | P value |
| --- | --- | --- | --- | --- | --- |
| Primary binary NRPC: Low vs High | 19/92 vs 18/164 | 2.11 (1.04–4.27) | 0.037 | 1.70 (0.80–3.57) | 0.165 |
| NRPC total score, per 1-point increase | 37/256 | 0.64 (0.45–0.92) | 0.015 | 0.70 (0.48–1.02) | 0.061 |
| Completed radiation-protection training within previous 12 months: Yes vs No | 21/190 vs 16/66 | 0.39 (0.19–0.80) | 0.010 | 0.44 (0.21–0.94) | 0.034 |
| No radiation-protection–related quality-control deductions within previous 12 months: Yes vs No | 19/160 vs 18/96 | 0.58 (0.29–1.18) | 0.133 | 0.67 (0.32–1.39) | 0.282 |
| Case-level documentation of radiation-protection measures: Yes vs No | 13/110 vs 24/146 | 0.68 (0.33–1.41) | 0.300 | 0.79 (0.37–1.67) | 0.538 |

NRPC, nursing radiation-protection compliance; OR, odds ratio; CI, confidence interval. Low NRPC was defined as a total score of 0–1 and High NRPC as a total score of 2–3. For the ordinal analysis, the OR represents the change in odds of the 30-day composite wound- and pin-site–related adverse event associated with each 1-point increase in NRPC score.

*Each adjusted model included calendar year of surgery, Gartland classification (type III/IV vs type II), operative duration (>60 vs ≤60 min), and emergency versus elective surgery. Each alternative NRPC representation was evaluated in a separate model. The three individual NRPC components were not entered simultaneously because they jointly constitute the composite NRPC score and are inherently correlated. Component-level analyses were exploratory and were undertaken to examine whether the overall pattern was dependent on a single component. These analyses were intended as internal robustness checks and should not be interpreted as formal validation of the NRPC construct.
