# Supplementary material for: Nursing radiation-protection compliance and 30-day wound and pin-site adverse events after supracondylar humerus fracture fixation in children
Source: Front Pediatr. 2026 Oct 15;14:1902962. doi: 10.3389/fped.2026.1902962 (PMC13716741; doi:10.3389/fped.2026.1902962)
Supplement: Supplementary file 2 [file Table2.docx]

**Supplementary Table S2. Temporal distribution of nursing radiation-protection compliance and 30-day wound- and pin-site–related adverse events during the study period**

| Calendar year | Patients, n | High NRPC, n/N (%) | Low NRPC, n/N (%) | Mean NRPC score | 30-day adverse events, n/N (%) |
| --- | --- | --- | --- | --- | --- |
| 2018 | 38 | 17/38 (44.7%) | 21/38 (55.3%) | 1.45 | 8/38 (21.1%) |
| 2019 | 40 | 21/40 (52.5%) | 19/40 (47.5%) | 1.53 | 7/40 (17.5%) |
| 2020 | 42 | 25/42 (59.5%) | 17/42 (40.5%) | 1.71 | 7/42 (16.7%) |
| 2021 | 44 | 29/44 (65.9%) | 15/44 (34.1%) | 1.84 | 6/44 (13.6%) |
| 2022 | 45 | 34/45 (75.6%) | 11/45 (24.4%) | 2.04 | 5/45 (11.1%) |
| 2023 | 47 | 38/47 (80.9%) | 9/47 (19.1%) | 2.11 | 4/47 (8.5%) |
| Total | 256 | 164/256 (64.1%) | 92/256 (35.9%) | 1.81 | 37/256 (14.5%) |

NRPC, nursing radiation-protection compliance. High NRPC was defined as a total score of 2–3 and Low NRPC as a total score of 0–1. The NRPC score ranged from 0 to 3 and was calculated from completion of recent formal radiation-protection training, absence of radiation-protection–related quality-control deductions, and case-level documentation of radiation-protection measures.

Calendar year was examined because both nursing compliance practices and broader perioperative processes could have changed during the multiyear study period. The temporal pattern was therefore treated as a potential source of secular confounding, and calendar year was included in the parsimonious adjusted and Firth penalized logistic regression models. Annual data are descriptive and should not be interpreted as demonstrating a causal effect of calendar time on either NRPC or postoperative outcomes.
