# Supplementary material for: Nursing radiation-protection compliance and 30-day wound and pin-site adverse events after supracondylar humerus fracture fixation in children
Source: Front Pediatr. 2026 Oct 15;14:1902962. doi: 10.3389/fped.2026.1902962 (PMC13716741; doi:10.3389/fped.2026.1902962)
Supplement: Supplementary file 3 [file Table3.docx]

**Supplementary Table S3. Provider-level case distribution and sensitivity analyses accounting for clustering by circulating nurse and primary surgeon**

**Panel A. Provider-level case distribution**

| Provider characteristic | Circulating nurse | Primary surgeon |
| --- | --- | --- |
| Unique providers, n | 18 | 12 |
| Total procedures contributed, n | 256 | 256 |
| Cases per provider, median | 13 | 20.5 |
| Cases per provider, IQR | 9–18 | 14–27 |
| Cases per provider, range | 5–24 | 7–35 |
| Intraclass correlation coefficient for primary outcome | 0.04 | 0.03 |

**Panel B. Sensitivity analyses of the Low versus High NRPC association**

| Analysis | OR for Low vs High NRPC (95% CI) | P value |
| --- | --- | --- |
| Crude logistic regression | 2.11 (1.04–4.27) | 0.037 |
| Parsimonious adjusted logistic regression | 1.70 (0.80–3.57) | 0.165 |
| Firth penalized logistic regression | 1.67 (0.80–3.49) | 0.168 |
| Mixed-effects logistic regression with circulating-nurse random intercept | 1.65 (0.76–3.58) | 0.205 |
| Mixed-effects logistic regression with primary-surgeon random intercept | 1.68 (0.77–3.66) | 0.190 |

NRPC, nursing radiation-protection compliance; OR, odds ratio; CI, confidence interval; IQR, interquartile range; ICC, intraclass correlation coefficient. Low NRPC was defined as a total score of 0–1 and High NRPC as a total score of 2–3. All adjusted models included calendar year of surgery, Gartland classification (type III/IV vs type II), operative duration (>60 vs ≤60 min), and emergency versus elective surgery. The nurse- and surgeon-clustered analyses were fitted as separate random-intercept mixed-effects logistic regression models using the circulating nurse and primary surgeon, respectively, as clustering factors. The ICCs quantify the estimated residual provider-level correlation in the corresponding models. These analyses were prespecified as sensitivity analyses and did not replace the primary parsimonious logistic regression model.
